# Supplementary material for: Mapping the evolution of fertility support policies in China: A content and instrumental analysis
Source: PLoS One. 2025 Oct 9;20(10):e0332137. doi: 10.1371/journal.pone.0332137 (PMC12510515; doi:10.1371/journal.pone.0332137)
Supplement: S1 Appendix — (ZIP) [file pone.0332137.s001.zip › S1 Appendix. 226 original policy documents/162-中华人民共和国母婴保健法实施办法(2001)(FBM-CLI-2-35752).doc]

中华人民共和国母婴保健法实施办法(2001)

**发布部门：** [国务院](https://www.pkulaw.com/law/chl?Aggs.IssueDepartment=5)

**发文字号：**中华人民共和国国务院令第308号

**发布日期：**2001.06.20

**实施日期：**2001.06.20

**时效性：** [已被修改](https://www.pkulaw.com/law/chl?Aggs.TimelinessDic=03)

**效力级别：** [行政法规](https://www.pkulaw.com/law/chl?Aggs.EffectivenessDic=XC0201)

**法规类别：** [妇幼卫生](https://www.pkulaw.com/law/chl?Aggs.Category=09622)

**修改依据：**[国务院关于修改部分行政法规的决定(国务院令第690号)](javascript:TMP(305664,0,'chl'))

**法宝提示：**本篇法规的变更情况请参考：[国务院关于第六批取消和调整行政审批项目的决定](javascript:TMP(185963,0,'chl'))

[2017-2022编注](https://www.pkulaw.com/history/compare/编注-4094.html) [2001-2017编注](https://www.pkulaw.com/history/compare/编注-1177.html) [1995-2001对照](https://www.pkulaw.com/history/compare/对照-1179.html)

中华人民共和国国务院令
（第３０８号）

　　现公布《中华人民共和国母婴保健法实施办法》，自公布之日起施行。
　　　　　　　　　　　　　　　　　　　　　　　　　　　　总理　朱镕基
　　　　　　　　　　　　　　　　　　　　　　　　　　２００１年６月２０日
　　　　　　　　　　中华人民共和国母婴保健法实施办法

第一章　总则

　　**第一条**　根据《[中华人民共和国母婴保健法](https://www.pkulaw.com/chl/ea633acd04298a40bdfb.html)》（以下简称[母婴保健法](https://www.pkulaw.com/chl/ea633acd04298a40bdfb.html)），制定本办法。

　　**第二条**　在中华人民共和国境内从事母婴保健服务活动的机构及其人员应当遵守[母婴保健法](https://www.pkulaw.com/chl/ea633acd04298a40bdfb.html)和本办法。

　　从事计划生育技术服务的机构开展计划生育技术服务活动，依照《[计划生育技术服务管理条例](https://www.pkulaw.com/chl/b160eb1e4abad895bdfb.html)》的规定执行。

　　**第三条**　母婴保健技术服务主要包括下列事项：

　　（一）有关母婴保健的科普宣传、教育和咨询；

　　（二）婚前医学检查；

　　（三）产前诊断和遗传病诊断；

　　（四）助产技术；

　　（五）实施医学上需要的节育手术；

　　（六）新生儿疾病筛查；

　　（七）有关生育、节育、不育的其他生殖保健服务。

　　**第四条**　公民享有母婴保健的知情选择权。国家保障公民获得适宜的母婴保健服务的权利。

　　**第五条**　母婴保健工作以保健为中心，以保障生殖健康为目的，实行保健和临床相结合，面向群体、面向基层和预防为主的方针。

　　**第六条**　各级人民政府应当将母婴保健工作纳入本级国民经济和社会发展计划，为母婴保健事业的发展提供必要的经济、技术和物质条件，并对少数民族地区、贫困地区的母婴保健事业给予特殊支持。

　　县级以上地方人民政府根据本地区的实际情况和需要，可以设立母婴保健事业发展专项资金。

　　**第七条**　国务院卫生行政部门主管全国母婴保健工作，履行下列职责：

　　（一）制定[母婴保健法](https://www.pkulaw.com/chl/ea633acd04298a40bdfb.html)及本办法的配套规章和技术规范；

　　（二）按照分级分类指导的原则，制定全国母婴保健工作发展规划和实施步骤；

　　（三）组织推广母婴保健及其他生殖健康的适宜技术；

　　（四）对母婴保健工作实施监督。

　　**第八条**　县级以上各级人民政府财政、公安、民政、教育、劳动保障、计划生育等部门应当在各自职责范围内，配合同级卫生行政部门做好母婴保健工作。

第二章　婚前保健

　　**第九条**　[母婴保健法](https://www.pkulaw.com/chl/ea633acd04298a40bdfb.html)第[七条](https://www.pkulaw.com/chl/ea633acd04298a40bdfb.html#tiao_7)所称婚前卫生指导，包括下列事项：

　　（一）有关性卫生的保健和教育；

　　（二）新婚避孕知识及计划生育指导；

　　（三）受孕前的准备、环境和疾病对后代影响等孕前保健知识；

　　（四）遗传病的基本知识；

　　（五）影响婚育的有关疾病的基本知识；

　　（六）其他生殖健康知识。

　　医师进行婚前卫生咨询时，应当为服务对象提供科学的信息，对可能产生的结果进行指导，并提出适当的建议。

　　**第十条**　在实行婚前医学检查的地区，准备结婚的男女双方在办理结婚登记前，应当到医疗、保健机构进行婚前医学检查。

　　**第十一条**　从事婚前医学检查的医疗、保健机构，由其所在地设区的市级人民政府卫生行政部门进行审查；符合条件的，在其《医疗机构执业许可证》上注明。

　　**第十二条**　申请从事婚前医学检查的医疗、保健机构应当具备下列条件：

　　（一）分别设置专用的男、女婚前医学检查室，配备常规检查和专科检查设备；

　　（二）设置婚前生殖健康宣传教育室；

　　（三）具有符合条件的进行男、女婚前医学检查的执业医师。

　　**第十三条**　婚前医学检查包括询问病史、体格及相关检查。

　　婚前医学检查应当遵守[婚前保健工作规范](https://www.pkulaw.com/chl/050813520a2a148fbdfb.html)并按照婚前医学检查项目进行。[婚前保健工作规范](https://www.pkulaw.com/chl/050813520a2a148fbdfb.html)和婚前医学检查项目由国务院卫生行政部门规定。

　　**第十四条**　经婚前医学检查，医疗、保健机构应当向接受婚前医学检查的当事人出具婚前医学检查证明。

　　婚前医学检查证明应当列明是否发现下列疾病：

　　（一）在传染期内的指定传染病；

　　（二）在发病期内的有关精神病；

　　（三）不宜生育的严重遗传性疾病；

　　（四）医学上认为不宜结婚的其他疾病。

　　发现前款第（一）项、第（二）项、第（三）项疾病的，医师应当向当事人说明情况，提出预防、治疗以及采取相应医学措施的建议。当事人依据医生的医学意见，可以暂缓结婚，也可以自愿采用长效避孕措施或者结扎手术；医疗、保健机构应当为其治疗提供医学咨询和医疗服务。

　　**第十五条**　经婚前医学检查，医疗、保健机构不能确诊的，应当转到设区的市级以上人民政府卫生行政部门指定的医疗、保健机构确诊。

　　**第十六条**　在实行婚前医学检查的地区，婚姻登记机关在办理结婚登记时，应当查验婚前医学检查证明或者[母婴保健法](https://www.pkulaw.com/chl/ea633acd04298a40bdfb.html)第[十一条](https://www.pkulaw.com/chl/ea633acd04298a40bdfb.html#tiao_11)规定的医学鉴定证明。

第三章　孕产期保健

　　**第十七条**　医疗、保健机构应当为育龄妇女提供有关避孕、节育、生育、不育和生殖健康的咨询和医疗保健服务。

　　医师发现或者怀疑育龄夫妻患有严重遗传性疾病的，应当提出医学意见；限于现有医疗技术水平难以确诊的，应当向当事人说明情况。育龄夫妻可以选择避孕、节育、不孕等相应的医学措施。

　　**第十八条**　医疗、保健机构应当为孕产妇提供下列医疗保健服务：

　　（一）为孕产妇建立保健手册（卡），定期进行产前检查；

　　（二）为孕产妇提供卫生、营养、心理等方面的医学指导与咨询；

　　（三）对高危孕妇进行重点监护、随访和医疗保健服务；

　　（四）为孕产妇提供安全分娩技术服务；

　　（五）定期进行产后访视，指导产妇科学喂养婴儿；

　　（六）提供避孕咨询指导和技术服务；

　　（七）对产妇及其家属进行生殖健康教育和科学育儿知识教育；

　　（八）其他孕产期保健服务。

　　**第十九条**　医疗、保健机构发现孕妇患有下列严重疾病或者接触物理、化学、生物等有毒、有害因素，可能危及孕妇生命安全或者可能严重影响孕妇健康和胎儿正常发育的，应当对孕妇进行医学指导和下列必要的医学检查：

　　（一）严重的妊娠合并症或者并发症；

　　（二）严重的精神性疾病；

　　（三）国务院卫生行政部门规定的严重影响生育的其他疾病。

　　**第二十条**　孕妇有下列情形之一的，医师应当对其进行产前诊断：

　　（一）羊水过多或者过少的；

　　（二）胎儿发育异常或者胎儿有可疑畸形的；

　　（三）孕早期接触过可能导致胎儿先天缺陷的物质的；

　　（四）有遗传病家族史或者曾经分娩过先天性严重缺陷婴儿的；

　　（五）初产妇年龄超过３５周岁的。

　　**第二十一条**　[母婴保健法](https://www.pkulaw.com/chl/ea633acd04298a40bdfb.html)第[十八条](https://www.pkulaw.com/chl/ea633acd04298a40bdfb.html#tiao_18)规定的胎儿的严重遗传性疾病、胎儿的严重缺陷、孕妇患继续妊娠可能危及其生命健康和安全的严重疾病目录，由国务院卫生行政部门规定。

　　**第二十二条**　生育过严重遗传性疾病或者严重缺陷患儿的，再次妊娠前，夫妻双方应当按照国家有关规定到医疗、保健机构进行医学检查。医疗、保健机构应当向当事人介绍有关遗传性疾病的知识，给予咨询、指导。对诊断患有医学上认为不宜生育的严重遗传性疾病的，医师应当向当事人说明情况，并提出医学意见。

　　**第二十三条**　严禁采用技术手段对胎儿进行性别鉴定。

　　对怀疑胎儿可能为伴性遗传病，需要进行性别鉴定的，由省、自治区、直辖市人民政府卫生行政部门指定的医疗、保健机构按照国务院卫生行政部门的规定进行鉴定。

　　**第二十四条**　国家提倡住院分娩。医疗、保健机构应当按照国务院卫生行政部门制定的技术操作规范，实施消毒接生和新生儿复苏，预防产伤及产后出血等产科并发症，降低孕产妇及围产儿发病率、死亡率。

　　没有条件住院分娩的，应当由经县级地方人民政府卫生行政部门许可并取得家庭接生员技术证书的人员接生。

　　高危孕妇应当在医疗、保健机构住院分娩。

第四章　婴儿保健

　　**第二十五条**　医疗、保健机构应当按照国家有关规定开展新生儿先天性、遗传性代谢病筛查、诊断、治疗和监测。

　　**第二十六条**　医疗、保健机构应当按照规定进行新生儿访视，建立儿童保健手册（卡），定期对其进行健康检查，提供有关预防疾病、合理膳食、促进智力发育等科学知识，做好婴儿多发病、常见病防治等医疗保健服务。

　　**第二十七条**　医疗、保健机构应当按照规定的程序和项目对婴儿进行预防接种。

　　婴儿的监护人应当保证婴儿及时接受预防接种。

　　**第二十八条**　国家推行母乳喂养。医疗、保健机构应当为实施母乳喂养提供技术指导，为住院分娩的产妇提供必要的母乳喂养条件。

　　医疗、保健机构不得向孕产妇和婴儿家庭宣传、推荐母乳代用品。

　　**第二十九条**　母乳代用品产品包装标签应当在显著位置标明母乳喂养的优越性。

　　母乳代用品生产者、销售者不得向医疗、保健机构赠送产品样品或者以推销为目的有条件地提供设备、资金和资料。

　　**第三十条**　妇女享有国家规定的产假。有不满１周岁婴儿的妇女，所在单位应当在劳动时间内为其安排一定的哺乳时间。

第五章　技术鉴定

　　**第三十一条**　母婴保健医学技术鉴定委员会分为省、市、县三级。

　　母婴保健医学技术鉴定委员会成员应当符合下列任职条件：

　　（一）县级母婴保健医学技术鉴定委员会成员应当具有主治医师以上专业技术职务；

　　（二）设区的市级和省级母婴保健医学技术鉴定委员会成员应当具有副主任医师以上专业技术职务。

　　**第三十二条**　当事人对婚前医学检查、遗传病诊断、产前诊断结果有异议，需要进一步确诊的，可以自接到检查或者诊断结果之日起１５日内向所在地县级或者设区的市级母婴保健医学技术鉴定委员会提出书面鉴定申请。

　　母婴保健医学技术鉴定委员会应当自接到鉴定申请之日起３０日内作出医学技术鉴定意见，并及时通知当事人。

　　当事人对鉴定意见有异议的，可以自接到鉴定意见通知书之日起１５日内向上一级母婴保健医学技术鉴定委员会申请再鉴定。

　　**第三十三条**　母婴保健医学技术鉴定委员会进行医学鉴定时须有５名以上相关专业医学技术鉴定委员会成员参加。

　　鉴定委员会成员应当在鉴定结论上署名；不同意见应当如实记录。鉴定委员会根据鉴定结论向当事人出具鉴定意见书。

　　[母婴保健医学技术鉴定管理办法](https://www.pkulaw.com/chl/0469d3e38f322315bdfb.html)由国务院卫生行政部门制定。

第六章　监督管理

　　**第三十四条**　县级以上地方人民政府卫生行政部门负责本行政区域内的母婴保健监督管理工作，履行下列监督管理职责：

　　（一）依照[母婴保健法](https://www.pkulaw.com/chl/ea633acd04298a40bdfb.html)和本办法以及国务院卫生行政部门规定的条件和技术标准，对从事母婴保健工作的机构和人员实施许可，并核发相应的许可证书；

　　（二）对[母婴保健法](https://www.pkulaw.com/chl/ea633acd04298a40bdfb.html)和本办法的执行情况进行监督检查；

　　（三）对违反[母婴保健法](https://www.pkulaw.com/chl/ea633acd04298a40bdfb.html)和本办法的行为，依法给予行政处罚；

　　（四）负责母婴保健工作监督管理的其他事项。

　　**第三十五条**　从事遗传病诊断、产前诊断的医疗、保健机构和人员，须经省、自治区、直辖市人民政府卫生行政部门许可。

　　从事婚前医学检查的医疗、保健机构和人员，须经设区的市级人民政府卫生行政部门许可。

　　从事助产技术服务、结扎手术和终止妊娠手术的医疗、保健机构和人员以及从事家庭接生的人员，须经县级人民政府卫生行政部门许可，并取得相应的合格证书。

　　**第三十六条**　卫生监督人员在执行职务时，应当出示证件。

　　卫生监督人员可以向医疗、保健机构了解情况，索取必要的资料，对母婴保健工作进行监督、检查，医疗、保健机构不得拒绝和隐瞒。

　　卫生监督人员对医疗、保健机构提供的技术资料负有保密的义务。

　　**第三十七条**　医疗、保健机构应当根据其从事的业务，配备相应的人员和医疗设备，对从事母婴保健工作的人员加强岗位业务培训和职业道德教育，并定期对其进行检查、考核。

　　医师和助产人员（包括家庭接生人员）应当严格遵守有关技术操作规范，认真填写各项记录，提高助产技术和服务质量。

　　助产人员的管理，按照国务院卫生行政部门的规定执行。

　　从事母婴保健工作的执业医师应当依照[母婴保健法](https://www.pkulaw.com/chl/ea633acd04298a40bdfb.html)的规定取得相应的资格。

　　**第三十八条**　医疗、保健机构应当按照国务院卫生行政部门的规定，对托幼园、所卫生保健工作进行业务指导。

　　**第三十九条**　国家建立孕产妇死亡、婴儿死亡和新生儿出生缺陷监测、报告制度。

第七章　罚则

　　**第四十条**　医疗、保健机构或者人员未取得母婴保健技术许可，擅自从事婚前医学检查、遗传病诊断、产前诊断、终止妊娠手术和医学技术鉴定或者出具有关医学证明的，由卫生行政部门给予警告，责令停止违法行为，没收违法所得；违法所得５０００元以上的，并处违法所得３倍以上５倍以下的罚款；没有违法所得或者违法所得不足５０００元的，并处５０００元以上２万元以下的罚款。

　　**第四十一条**　从事母婴保健技术服务的人员出具虚假医学证明文件的，依法给予行政处分；有下列情形之一的，由原发证部门撤销相应的母婴保健技术执业资格或者医师执业证书：

　　（一）因延误诊治，造成严重后果的；

　　（二）给当事人身心健康造成严重后果的；

　　（三）造成其他严重后果的。

　　**第四十二条**　违反本办法规定进行胎儿性别鉴定的，由卫生行政部门给予警告，责令停止违法行为；对医疗、保健机构直接负责的主管人员和其他直接责任人员，依法给予行政处分。进行胎儿性别鉴定两次以上的或者以营利为目的进行胎儿性别鉴定的，并由原发证机关撤销相应的母婴保健技术执业资格或者医师执业证书。

第八章　附则

　　**第四十三条**　婚前医学检查证明的格式由国务院卫生行政部门规定。

　　**第四十四条**　[母婴保健法](https://www.pkulaw.com/chl/ea633acd04298a40bdfb.html)及本办法所称的医疗、保健机构，是指依照《[医疗机构管理条例](https://www.pkulaw.com/chl/636b7756c3fa849abdfb.html)》取得卫生行政部门医疗机构执业许可的各级各类医疗机构。

　　**第四十五条**　本办法自公布之日起施行。

© 北大法宝：（[www.pkulaw.com](https://www.pkulaw.com/)) 专业提供法律信息、法学知识和法律软件领域各类解决方案。北大法宝为您提供丰富的参考资料，正式引用法规条文时请与标准文本核对。
欢迎查看所有[产品和服务](https://www.pkulaw.com/helps/2.html)。法宝快讯：[如何快速找到您需要的检索结果？](https://www.pkulaw.com/helps/70.html)[法宝](https://www.pkulaw.com/helps/69.html)V6有何新特色？

扫描二维码阅读原文

**原文链接：**<https://www.pkulaw.com/chl/5b028eec77f882eebdfb.html>
